# Optical Sensing of Weed Infestations at Harvest

**DOI:** 10.3390/s17102381

**Published:** 2017-10-19

**Authors:** Judit Barroso, John McCallum, Dan Long

**Affiliations:** 1 Columbia Basin Agricultural Research Center, Oregon State University, Adams, OR 97810, USA; 2 Soil and Water Conservation Research Unit, Agricultural Research Service (USDA-ARS), Adams, OR 97810, USA; John.McCallum@ars.usda.gov (J.M.); Dan.Long@ars.usda.gov (D.L.)

**Keywords:** weed mapping, on-line optical sensing, site-specific weed management

## Abstract

Kochia (*Kochia scoparia* L.), Russian thistle (*Salsola* *tragus* L.), and prickly lettuce (*Lactuca serriola* L.) are economically important weeds infesting dryland wheat (*Triticum aestivum* L.) production systems in the western United States. Those weeds produce most of their seeds post-harvest. The objectives of this study were to determine the ability of an optical sensor, installed for on-the-go measurement of grain protein concentration, to detect the presence of green plant matter in flowing grain and assess the potential usefulness of this information for mapping weeds at harvest. Spectra of the grain stream were recorded continuously at a rate of 0.33 Hz during harvest of two spring wheat fields of 1.9 and 5.4 ha. All readings were georeferenced using a Global Positioning System (GPS) receiver with 1 m positional accuracy. Chlorophyll of green plant matter was detectable in the red (638–710 nm) waveband. Maps of the chlorophyll signal from both fields showed an overall agreement of 78.1% with reference maps, one constructed prior to harvest and the other at harvest time, both based on visual evaluations of the three green weed species conducted by experts. Information on weed distributions at harvest may be useful for controlling post-harvest using variable rate technology for herbicide applications.

## 1. Introduction

Weeds are often distributed in farm fields as many irregular patches [1,2] that are relatively stable from one season to the next [3,4,5,6]. Consequently, some growers have been motivated to consider site-specific weed management (SSWM) to apply herbicides only to sites within fields that are weed infested. Site-specific weed management has been shown to offer economic and environmental benefits [7,8]. Before growers can benefit from SSWM, they must know where weeds are located within their fields. Today, modern spray delivery systems can be controlled either (i) indirectly from a weed map derived from remotely sensed information that had been acquired at an earlier time [9], or (ii) directly in response to the outputs from a sensing system mounted on the treatment vehicle [10,11]. These technologies rely upon machine vision systems capable of detecting green plants and discriminating weeds from the crop.

Low altitude aerial- and ground-based sensing systems are being emphasized that can provide data for detecting weeds between crop rows [12,13]. For example, both the spectral reflectance characteristic of vegetation and the inter-row position of weeds were used to distinguish weeds from the row position of the crop [14]. Similarly, weeds emerging between rows and within rows early in the season were detected using object-based image analysis of radiometric, spatial, geometric, and textural characteristics in high resolution (1.4 cm), true-color digital imagery obtained from an unmanned aerial vehicle (UAV) at 30 m above ground level [15]. Other sensor technologies under development include spectrometric-optoelectronic sensors to differentiate plants from soil [16,17], fluorescence sensors to discriminate plant species [18], and stereovision and depth cameras to characterize plant structure [19,20].

Results of research are sufficiently promising to suggest that weed detection using machine vision is feasible [21,22,23,24], but no commercial products are in place at an affordable cost. For spring SSWM, an early-season weed detection method is ideal. However, for weed species that either germinate or produce seeds late in the season that are mostly a problem post-harvest, late-season detection methods could be suitable as well. An advantage of sensing at this time is that the probability of confusing weeds with the crop decreases.

Spectrometers are commercially available that are capable of measuring and mapping the protein concentration of grain while harvesting. These spectrometers measure reflected (or transmitted) light within the visible and near-infrared (NIR) portions of the electromagnetic spectrum. Grain protein content can be calculated from hundreds of scans averaged over a few seconds corresponding to measurement rates of 100 Hz (10 ms per scan). When georeferenced with a GPS receiver, grain protein measurements can be produced at relatively high spatial resolution as determined by harvesting width and speed.

Kochia, Russian thistle, and prickly lettuce have been ranked among the ten most troublesome weeds in dryland cereal grain crops of the western United States [25]. The annual cost for control of Russian thistle has been estimated at more than US $50 M in states of Oregon, Washington, and Idaho [26]. In those states, prickly lettuce was found to reduce grain yields by up to 25% in spring wheat [27] and kochia up to 30% in wheat [28]. These three weed species are late maturing weeds that are often green at time of wheat harvest when the crop has senesced. Green material from weeds is ground up by the combine’s cylinder and some of it is picked up with the shelled kernels of grain. The spectral reflectance of this green material provides information about the amount of chlorophyll present, which can be used to indicate the presence of weeds. No reports have been found in the literature in which weeds were mapped by optically sensing the presence of green foreign material in the grain stream during harvest. The objectives of this study were to apply on-combine grain spectroscopy into mapping the spatial distribution of late-season weeds and assess the potential usefulness of this information for SSWM.

## 2. Materials and Methods

### 2.1. Study Site

On-combine optical weed sensing was evaluated in two spring wheat fields that had infestations of kochia, Russian thistle and prickly lettuce in 2015. Kochia and Russian thistle are summer annual species and prickly lettuce is an annual, winter annual, or occasionally a biennial species. Field I (1.9 ha) and Field II (5.4 ha) are located at the Oregon State University Columbia Basin Agricultural Research Center (45°43.7′ N. Lat.; 118°37.4′ W Lon.) in eastern Oregon, USA. Both fields were direct seeded with a crop density of about 270 seed m^−2^ and a row spacing of 25 cm. Fertilizer was applied before and during seeding using liquid formulation at pre-seeding (90 kg N ha^−1^) and granules at seeding (14.5 kg of N ha^−1^ and 22 kg of P_2_O_2_ ha^−1^). Both fields also received a glyphosate application (at 420 g ae ha^−1^) before seeding for weed control. The climate is characterized by 425 mm average annual precipitation of which 70% occurs during the fall and winter months (23 September–20 March). Summers are dry and hot with maximum temperatures reaching over 38 °C.

### 2.2. Acquisition of Spectroscopic Data

**Figure 1:**
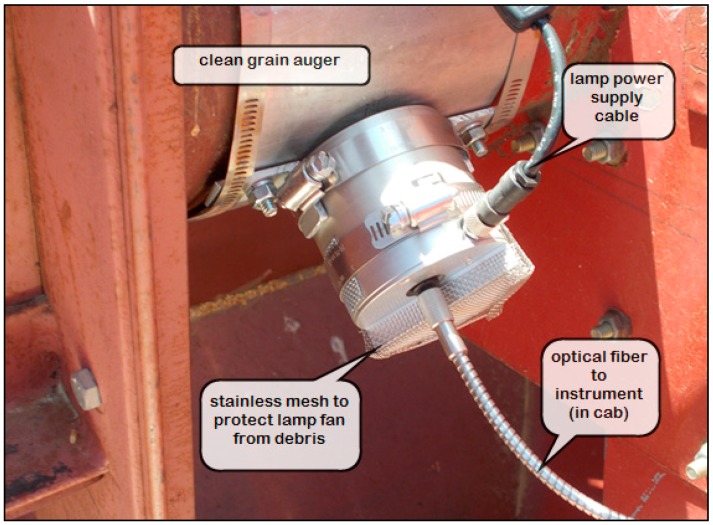
Sensor head and fiber optic cable of the Avaspec model 2048 spectrometer mounted to the combine’s grain bin filling auger to measure grain protein and detect green plant material in the harvested grain.

In this study, the AvaSpec ULS2048RS (Avantes, Apeldoorn, The Netherlands) spectrometer was evaluated for measuring the presence of chlorophyll in the grain stream of wheat during harvest with a combine. The instrument consists of three components: (i) a fiber optic probe; (ii) an electronic spectrometer unit; and (iii) a computer processing unit (CPU) with the instrument control software (Avantes AvaSoft-Basic). The sensor head (70 mm dia.) was mounted to the housing of the grain bin filling auger of a Case International Harvester (IH) 1470 combine (Figure 1). The head’s 2 m fiber optic cable was long enough to reach the spectrometer unit and CPU that were placed inside the combine’s cab. A Durabook model R150D notebook computer was used as the CPU for running the instrument and collecting spectral data. An inverter provided the computer 120 VAC power from an independent 12 VDC lead-acid battery. In turn, the spectrometer and sensor head ran on 0.3 A of 5 VDC power through the computer’s USB port.

The AvaSpec instrument measures diffuse reflectance spectra in 0.22–0.28 nm intervals over a wavelength range from 638 to 1163 nm. The spectrometer has a detector that is a silicon-based Charged Coupled Device linear array of 2048 photo-diode elements. The sensor head at the end of the fiber optic cable is a combined integrating sphere and halogen light source (Avantes AvaSphere-50-LS-HAL) that illuminates the sample and collects the returning reflectance signal. The fiber optic cable transmits the reflected spectra from the sensor head to the detector in the spectrometer unit at a rate of 15 spectra minute^−1^. A white reference scan of a 99% Spectralon^®^ Diffuse Reference Standard (Labsphere Inc., North Sutton, NH, USA) was taken manually at the beginning of each day as needed to calibrate the sensor. Field I was harvested on 23 July 2015 and Field II on 27 July 2015.

### 2.3. Acquisition of Reference Weed Data

Two reference sampling methods were used to determine the accuracy of the weed map produced by means of the on-combine sensing method:

1. Weed mapping by visual evaluation from the ground. Prior to harvest, a handheld computer with built-in GPS receiver (Yuma2^®^ Rugged Tablet Computer, Trimble, Sunnyvale, CA, USA) was used to partition the field into numerous 7 m × 7 m cells. Each cell was encountered along a series of parallel transects spaced 7 m apart to match combine header width and position to weed location. Weed abundance per species in four categories of plant density were coded as follows: none (0), low (1), moderate (2), and high (3), were recorded every 7 m along each transect.
2. Weed mapping by visual evaluation from the combine. During harvest, the handheld computer with built-in GPS receiver was used from the cab of the combine to code weed abundance of green species in the same 7 m × 7 m cells as described in method 1 above. The combine’s ground speed varied between 4 and 6 km h^−1^ depending on weed infestation and crop density.

### 2.4. Data Processing

Raw spectra were cleaned of erroneous values: e.g., measurements registered at an empty header. Out of range values were identified using histograms and eliminated using the spreadsheet software MS-Excel. Position errors resulting from start and stop delays for beginning and ending of passes were determined by plotting the data series versus the Coordinated Universal Time recorded by the GPS receiver and noting the time differential between structural features of data associated with certain geographical features of the field. A time delay of 18 s was applied to shift the grain spectra from GPS points where spectra were read at the grain bin filling auger to the GPS points where the wheat was cut.

**Figure 2:**
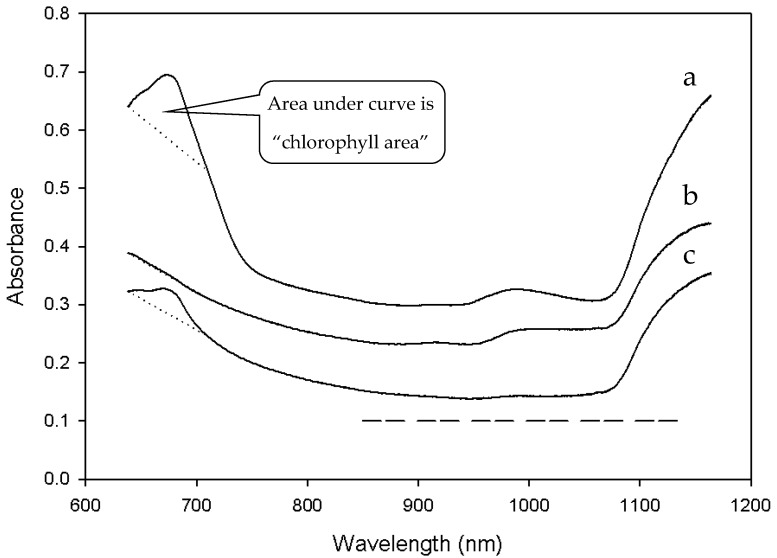
Illustration of spectral absorbance versus wavelength for grain with high (spectrum of line ‘a’), null (spectrum of line ‘b’) and low (spectrum of line ‘c’) amounts of foreign, green weedy matter. Areas under spectra from 638 nm and 710 nm above the baseline (dotted line) are “chlorophyll area” where green weedy matter in the grain stream is detectable. Also shown dashed line is the analysis region where protein concentration of grain is detectable.

Reflectance spectra of grain kernels vary with distribution and amount of protein within the endosperm. Foreign material from green plants deepens the absorbance feature in the red waveband. The wavelengths used in protein analysis do not overlap with the chlorophyll peak. Accordingly, grain with green foreign material was detected by the “chlorophyll area” defined as the area below the spectral signature and above a linear baseline from 638 nm and 710 nm, where a chlorophyll peak was easily identifiable (Figure 2).

### 2.5. Statistical Analyses

Geographic coordinates of the spectroscopic and reference data were projected to UTM coordinates using the ArcGIS^®^ software version 10.4.1 (ESRI, Redlands, CA, USA). To compare the methods, data from the two reference methods and the spectrometer were interpolated to a common 7 m × 7 m grid using the modified Shepard’s method [29] with the equation indicated below:

$$f\left(x_{q}\right)=\frac{\sum_{i=1}^{k}w_{i}*f\left(x_{i}\right)}{\sum_{i=1}^{k}w_{i}}\;\mathrm{with}\;w_{i}=\frac{1}{d{\left(x_{q},\;x_{i}\right)}^{2}}$$

where *f(x_q_)* is the new value (interpolated value), *f(x_i_)* is the original data value, and *w_i_* are the weights (*d(x_q_,x_i_)* as a function of distance (m) between the location of the interpolated point and original data).

**Figure 3:**
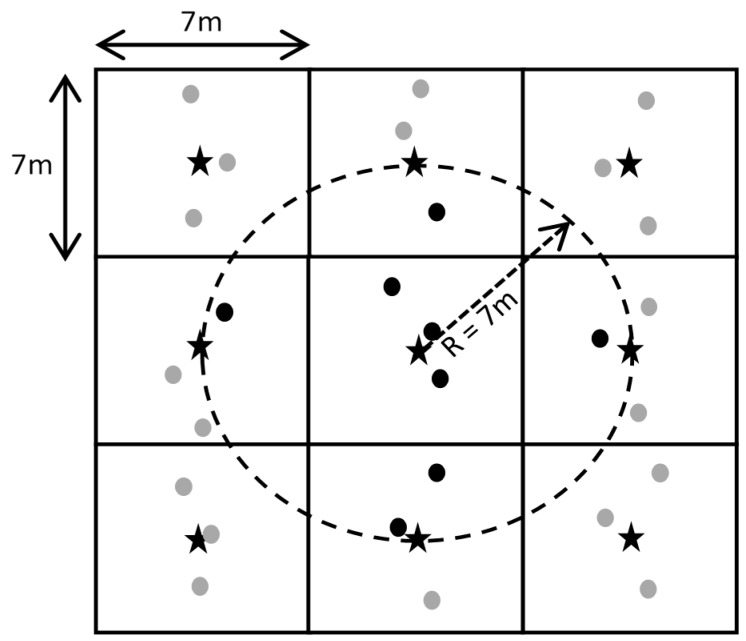
Interpolation of points on a 7 m × 7 m grid. Black stars indicate position of interpolated values at the nodes of a 7 m grid, dashed circle indicates an R-sphere with 7 m radius, black dots indicate positions of experimental data used to estimate the value of the central node, and grey dots indicate positions of experimental data beyond the R-sphere and not used.

**Figure 4:**
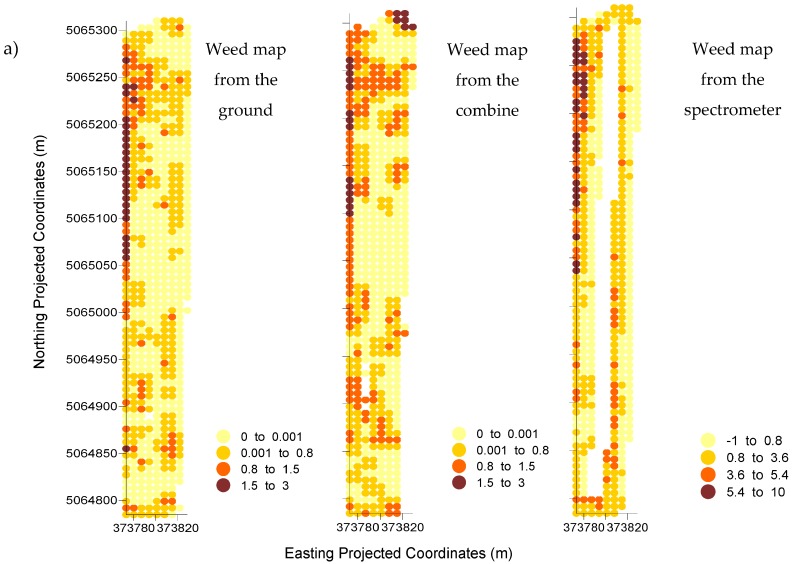

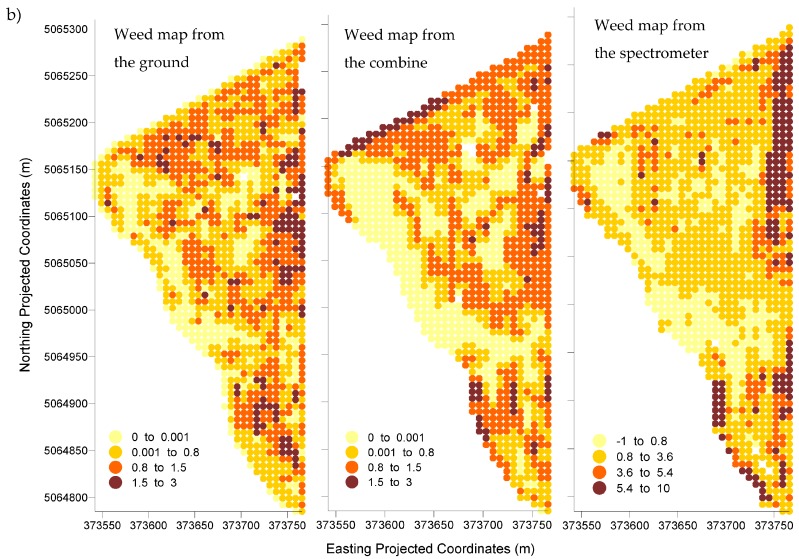
Infestation maps of green weeds at harvest of Field I (**a**), and Field II (**b**). Note: White areas inside the fields are missing data. The software used to build the maps was Surfer^®^ version 8.0.

An R-sphere of 7 m was used to identify the neighborhood about each node on the grid (Figure 3). For reference data, a weighted value was taken of the integer values (0, 1, 2 and 3) within a 7-m neighborhood such that the returned output consisted of ratio-interval values ranging from 0.0 to 3.0 as illustrated in Figure 4. Spectrometer chlorophyll areas were interpolated in the same way with output ranging from −1.0 to 10.0.

Interpolated values for weed density from reference methods 1 and 2 were used to divide Fields I and II into two zones of weeds present and weeds absent. Cutoff values were used to divide the distribution into three levels of zero (<0.001), low (<0.8), and moderate (<1.5) weed tolerance. Under zero tolerance, a lower limit produced two map classes: weeds absent (<0.001) and weeds present (≥0.001) and would be appropriate if one wanted to treat every weed. A low tolerance represented map classes of weeds absent (<0.8) or weeds present (≥0.8) as needed to capture weed densities below a level that could cause light economic loss. Moderate tolerance had map classes of weeds absent (<1.5) and weeds present (≥1.5) above a level that could cause economic yield loss.

**Figure 5:**
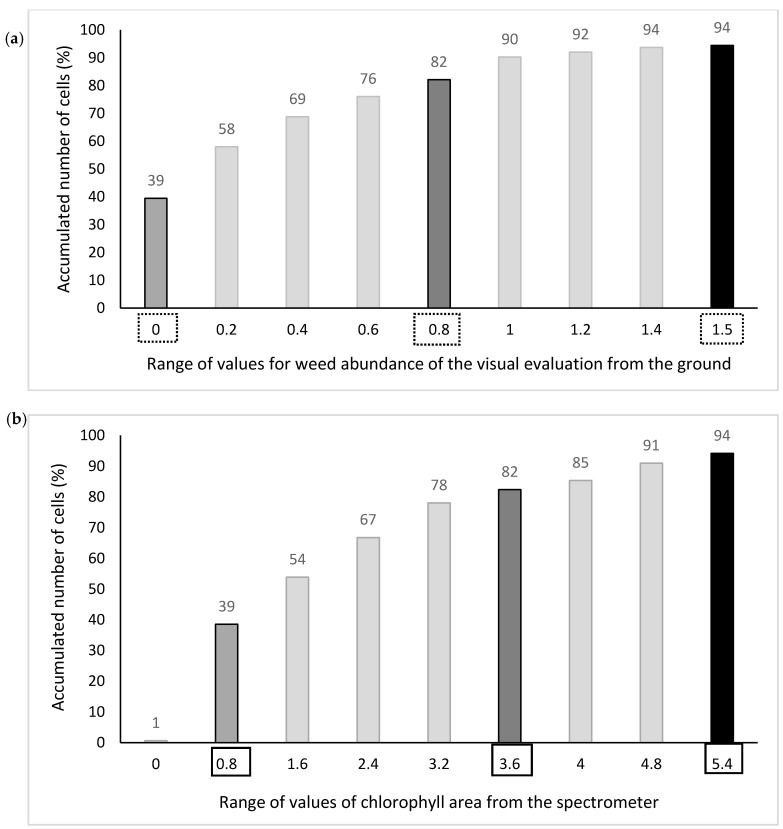
Histograms of the accumulated percentage of number of cells (7 m × 7 m) in the range of values of Field I: (**a**) for the visual weed evaluation from the ground (method 1), and (**b**) for the weed map from the spectrometer. Note: Weed tolerance thresholds for reference methods 1 and 2 are squared with a dotted black line. The three selected threshold values for the spectrometer are squared with a continued black line.

Fields I and II were divided in the same way as for interpolated spectrometer data except that cutoff values for the spectrometer were selected by noting where the cumulated percentages of reference method 1 in Field I coincided with those of the spectrometer method (Figure 5). The spectrometer values with matched percentage of accumulated cells for the different weed tolerance thresholds were considered the equivalent weed tolerance thresholds for the weed map from the spectrometer. An interpolated value of 0.8 in the reference map represented the average of a neighborhood of points that were mostly coded as 1 prior to interpolation. The corresponding cutoff value of 3.6 in the map derived from the spectrometer gave the same percentage of accumulated number of cells as the cutoff value of 0.8 in the reference map (Figure 5).

Using method 1 or 2 as a reference of comparison, confusion matrices were used to determine percent accuracy of the weed map derived from the spectrometer method as for each weed tolerance level. The diagonal of a confusion matrix indicated the percentage of cells correctly labeled as infested or non-infested between two maps (agreement). The off-diagonal cells indicated cells in disagreement between two maps. There were two types of errors, or disagreements, errors of omission (cells infested but not detected) and errors of commission (cells not infested but detected as infested). Map comparison was conducted with a minimum of 450 cells for Field I and 1290 cells for Field II.

## 3. Results

### 3.1. Comparison of Reference Maps

In Field I, the reference map derived from the visual evaluation of weeds from the ground had a 54.7, 14.7, and 4.5 percentage of infested cells for the zero, low, and moderate weed tolerance thresholds. In Field II, the percentage of infested cells was 83.9, 43.5, and 8.7 for the same order of weed tolerance thresholds. Percentages for the visual evaluation from the combine were 55.3, 24.2, and 3.5 in Field I and 73.2, 44.8, 6.0 in Field II. Both methods indicated that Field II had a higher percentage of its area infested regardless of weed tolerance threshold.

The confusion matrix comparing the weed maps derived from both reference methods showed a different percentage of agreement depending on the weed tolerance threshold (Table 1). On average, the percentage of agreement was 82.8% for Field I and 79.3% for Field II. These percentages increased to 95.8% for Field I and 88.5% for Field II when the weed tolerance threshold was moderate (considering infested cells when their value was >1.5). The areas clearly infested (moderate to high infestation) were well classified with both reference methods but the areas with lower infestation produced more disagreement. The percentage of omission and commission errors averaged about 12% for zero and low weed tolerance thresholds and nearly 4% for the moderate weed tolerance threshold.

### 3.2. Comparison of Each Reference Map with the Spectrometer

For the spectrometer-derived weed map of Field I, the percentage of infested cells was 61.4, 17.9, and 5.6 for the identified zero, low, and moderate thresholds. For Field II, those percentages were 79.3, 22.0, and 10.2 for the same zero, low, and moderate thresholds. Data from the spectrometer also indicated that Field II was more infested than Field I regardless the weed tolerance threshold.

The confusion matrix that compared the spectrometer-based weed map with each reference map showed a different percentage of agreement depending on the weed tolerance threshold (Table 2). On average, there was 81.5% and 75.4% agreement for Fields I and II between the weed map evaluated from the ground and derived from the spectrometer. It was 77.2% for Field I and 78.3% for Field II for the comparison between the weed map evaluated from the combine and the one derived from the spectrometer. The comparison between the spectrometer-derived weed map and both reference methods was higher for moderate weed tolerance thresholds (90.5% for Field I and 91.2% for Field II) and lower for low weed tolerance thresholds (73.8% for Field I and 69.9% for Field II). Moderately to highly infested areas were identified more accurately (90.9%) than low to zero level of weed tolerance (71.9%) with any of the reference maps.

Except for the comparison between the weed map from the ground and the spectrometer of Field I, where the percentage of omission and commission errors were coincidental, the commission and omission errors were dependent on the weed tolerance thresholds. For zero and moderate weed tolerance thresholds, the percentage of commission errors (10.6% on average) was slightly higher than the omission errors (7.8% on average). However, for low weed tolerance thresholds, the percentage of omission errors (24.9% on average) was higher than the commission errors (7.3% on average).

The percentage of agreement for Field II between the spectrometer map and either of the reference maps (75.4% and 78.3%) was similar to the ones obtained for Field I (81.5% and 77.2%). This indicated that the selected threshold values for spectrometer data in Field I were validated to identify different weed densities in Field II.

## 4. Discussion

On-combine spectroscopy was highly accurate in correctly classifying cells that were infested with green weeds when a moderate weed tolerance threshold was used as indicated by an overall percentage agreement of 90.9% compared with 90.5% for reference method 1 and 91.2% for reference method 2. Accordingly, the number of cells that were falsely classified (omission or commission errors) was low (9.1% on average). These results are sufficiently promising to suggest that on-combine grain spectroscopy may be used to map green weeds in dryland wheat fields at harvest. Small areas may exist within fields where wheat has not reached physiological maturity. Green kernels may be confused with weeds and contribute to commission error. Our study pertained to fields that are fully mature.

Omission errors would result in infested areas that would go untreated and commission errors would result in non-infested areas that would be treated. The cost of omission errors will depend on yield potential of a field, price of the crop, and percentage of yield loss. For a common yield potential in the region (4000 kg/ha), a current wheat price ($0.19/kg), and a yield loss of 10%, the omission error would cost $76/ha. The cost of commission error will depend mostly on the cost of the herbicide/s used. For the cost of a common herbicide used post-harvest ($14.8/ha) and a regular herbicide application cost ($6.2/ha), the commission error would cost $21/ha. With the considered assumptions, a major cost was associated with the omission error, when weeds are left untreated. In the comparison between the spectrometer-derived weed map and either reference weed maps, a low weed tolerance threshold produced omission errors than were lower than commission errors. An exception occurred in Field I for the comparison with the evaluation from the ground that was used to determine the threshold values and consequently had equal percentage of omission or commission errors. Extreme weed tolerance thresholds (zero and moderate weed infestations) did not result in either commission error or omission error being predominant. Growers should consider the cost associated with these two error types and the prevalence of one or the other when deciding what weed tolerance threshold to use. Further research is needed to determine whether the method would work as well in other environments.

The similarity between the comparisons of both reference methods with the spectrometer showed that the unlimited time to rate the presence of weeds in the visual evaluation from the ground compared with ratings given from a combine moving at 5 km/h (average speed), was compensated with the advantage of having a more elevated view. Previous work showed that estimating infestations from the combine was as accurate as traditional methods used by weed scientists in which weeds are sampled within small quadrats within the area to be evaluated [30].

A weed map provided by a low-cost VIS spectrometer such as the AvaSpec instrument is potentially useful in SSWM to direct post-harvest weed control practices within fields. Site-specific, late-season herbicide applications (after harvest) might offer larger benefits over early-season herbicide applications because herbicide doses tend to be higher and thus savings from areas left untreated would be greater. Doses are higher post-harvest in August because weeds are larger and environmental conditions are less appropriate for herbicide activity than in spring. However, using on-combine spectroscopy for mapping green weeds at harvest would require commercial software to automate data processing and produce weed maps.

Designed for controlling weeds in real-time, the commercial spot spray system WeedSeeker® has shown good potential for herbicide reduction [23,24], but the main disadvantage is that the required investment is still only affordable for a few growers. High cost is considered to be a leading factor preventing adoption of precision agriculture [31]. By relying upon a relatively inexpensive spectrometer (US $5000), the weed mapping approach used in this study might help accelerate adoption of SSWM. Using on-line spectroscopy for mapping grain quality at harvest [32] also gives double use to a single investment. In addition, an advantage of spot spraying based on an at-harvest weed map over post-harvest detection and treatment of weeds in real-time is the possibility of preparing the correct amount of herbicide based on knowledge of the size of the infested area.

Further studies on the population dynamics of Russian thistle, kochia, and prickly lettuce would be necessary to assess the use of weed maps from the previous season for SSWM in the following growing season. If those species were stable, the weed map generated at harvest could also be used for pre-emergence or post-emergence applications in the following season.

## Figures and Tables

**Table 1:** Confusion matrix of the two reference maps (evaluation from the ground and evaluation from the combine) for different thresholds for Fields I and II.

Threshold		Confusion Matrix for Field I				Confusion Matrix for Field II			
Zero			Evaluation from ground				Evaluation from ground		
weed level			True	False	Agreement		True	False	Agreement
of tolerance	Evaluation	True	40.7	14.6	71.4	True	68.3	4.9	79.4
	from combine	False	14.0	30.7		False	15.6	11.1	
Low			Evaluation from ground				Evaluation from ground		
weed level			True	False	Agreement		True	False	Agreement
of tolerance	Evaluation	True	10.0	14.2	81.1	True	29.2	15.6	70.1
	from combine	False	4.7	71.1		False	14.3	40.9	
Moderate			Evaluation from ground				Evaluation from ground		
weed level			True	False	Agreement		True	False	Agreement
of tolerance	Evaluation	True	1.9	1.6	95.8	True	1.6	4.4	88.5
	from combine	False	2.6	93.9		False	7.1	86.9	

**Table 2:** Confusion matrix of the weed map from the spectrometer with each of the two reference maps for three different thresholds and for Field I and Field II.

Threshold		Confusion Matrix for Field I				Confusion Matrix for Field II			
Zero			Evaluation from ground				Evaluation from ground		
weed level			True	False	Agreement		True	False	Agreement
of tolerance	Spectrometer	True	43.5	17.9	65.1	True	69.7	9.6	77.2
		False	17.0	21.6		False	13.2	7.4	
Low			Evaluation from ground				Evaluation from ground		
weed level			True	False	Agreement		True	False	Agreement
of tolerance	Spectrometer	True	9.9	8.0	84.1	True	14.1	7.9	63.4
		False	8.0	74.1		False	28.7	49.3	
Moderate			Evaluation from ground				Evaluation from ground		
weed level			True	False	Agreement		True	False	Agreement
of tolerance	Spectrometer	True	3.2	2.4	95.3	True	2.2	8.0	85.7
		False	2.4	92.0		False	6.3	83.5	
Zero			Evaluation from combine				Evaluation from combine		
weed level			True	False	Agreement		True	False	Agreement
of tolerance	Spectrometer	True	43.7	21.6	64.0	True	66.7	12.8	80.5
		False	14.4	20.2		False	6.6	13.8	
Low			Evaluation from combine				Evaluation from combine		
weed level			True	False	Agreement		True	False	Agreement
of tolerance	Spectrometer	True	10.9	8.4	74.2	True	16.4	5.7	65.6
		False	17.4	63.3		False	28.7	49.2	
Moderate			Evaluation from combine				Evaluation from combine		
weed level			True	False	Agreement		True	False	Agreement
of tolerance	Spectrometer	True	2.1	4.0	93.5	True	2.6	7.7	88.8
		False	2.5	91.4		False	3.5	86.3	
